# Video-Based Simulation Among Saudi Undergraduate Nursing Students During COVID-19: A Qualitative Study

**DOI:** 10.7759/cureus.48605

**Published:** 2023-11-10

**Authors:** Yasir S Alsalamah, Maryam S Alharbi, Safiah Labani, Reham A Abed, Awadh S Al Harbi, Esin Kavuran, Nihan Türkoğlu, Hanan Al-Nuqaidan, Mirna Fawaz

**Affiliations:** 1 Department of Nursing, Mental Health Hospital, Qassim Health Cluster, Qassim, SAU; 2 Department of Nursing, University of Pretoria, Pretoria, ZAF; 3 Department of Pediatric Oncology & Hematology, King Abdullah Specialized Children's Hospital, Riyadh, SAU; 4 Research Unit, College of Dentistry, King Saud Bin Abdulaziz University for Health Sciences, Riyadh, SAU; 5 Medical and Surgical Ward, King Saud Medical City, Riyadh, SAU; 6 Department of Nursing, King Saud Hospital, Riyadh, SAU; 7 Department of Nursing, Ataturk University, Erzurum, TUR; 8 Department of Public Health Nursing, Ataturk University, Erzurum, TUR; 9 Department of Nursing, Ministry of Health, Kuwait, KWT; 10 Department of Nursing, Beirut Arab University, Beirut, LBN

**Keywords:** covid-19, nursing students, video-based learning, nursing education, simulation

## Abstract

Background

Because of university closures due to COVID-19 confinement, video-based simulation, a training technique based on high-fidelity simulations, was introduced in reaction to the need to adapt high-fidelity clinical simulation experiences to digital platforms.

Purpose

This study aims to evaluate the perceptions of nursing students in Saudi Arabia regarding the shift from face-to-face simulation experiences to video-based simulation during the COVID-19 pandemic.

Methods

This study employed a phenomenological exploratory qualitative research design among 32 nursing students from various academic levels.

Results

The thematic analysis gave rise to five themes namely, “Enhanced Learning and satisfaction”, “Improved communication skills”, “Lack of hands-on experience”, “More comfortable experience”, and “Technical Barriers”.

Conclusion

The students in this study have indicated that they were satisfied with video-based simulation, where they reported enhanced learning, better communication skills, and more perceived comfort, while concerns regarding technical issues and nursing skills were raised.

## Introduction

Introduction

The global pandemic caused by SARS-CoV-2 forced numerous governments to control social distancing with initiatives that started in March 2020. This involved contested policies such as the closing of schools and colleges to keep the population confined. This has raised significant challenges for academics all around the world, where the introduction of public health initiatives aiming at restricting human interaction to slow the virus's dissemination has led to the suspension of face-to-face learning and resorting to exclusive online learning techniques even in practical health majors such as nursing [1].

Nursing is considered one of the health-related majors that highly depend on hands-on experiences, critical thinking, knowledge, and skills fostered by experiences such as clinical placement and definitely clinical simulation experiences at the university in order to prepare the students and graduates for such complex clinical practice. Clinical simulation is, without a doubt, an important part of the nursing experience [2]. Face-to-face immersive learning environments with a simulator, a mannequin, a standard patient, and students make up a traditional simulation experience [3]. Yet, nursing students were deprived of the needed clinical preparations due to the applied restrictions, which have rendered the students frustrated about their skills and have incentivized the educators to look for replacements. This shifted the attention to emerging technologies, which might make it possible to extend simulation-based instruction to this unique circumstance. Where face-to-face simulation is not available, technical advancements and emerging technology might offer learners a relatively close, immersive virtual simulation learning environment through virtual media, apps, or mobile devices [4-5].

A pioneering simulation framework that re-creates high-fidelity experiences through virtual video-based scenarios tailored around various tasks and practices for nursing care is suggested in response to the need for simulation-based education triggered by restrictions set by the COVID-19 pandemic. This teaching approach has been shown to be a successful instrument for assessing students' qualifications and clinical outputs, and it is an important part of their preparation and education [6]. Furthermore, video-based simulation appeared to be an ideal way for students to prepare and adapt to this rapidly growing healthcare modality [7]. The introduction of this methodology would fill the current clinical gap in nursing education which is increasingly affecting the preservation and development of skills and competencies among nursing graduates, and thus undermining the quality of nursing care rendered to patients after graduation [6].

It was important to qualitatively evaluate the introduction of video-based simulation learning experiences as the employment of such an interactive new learning modality would entail a different experience for each student driven by their diverse perceptions of the educational process. The alignment with the qualitative paradigm will access the students’ worldview and provide nuanced experiences upon which nursing educators could design, develop, and improve technologically enhanced simulation experiences. Therefore, this study aims to explore the lived experiences of nursing students at one university in Saudi Arabia regarding the shift from face-to-face simulation experiences to video-based simulation during the COVID-19 pandemic.

## Materials and methods

Methodology

Research Design

The study utilized a phenomenological exploratory qualitative analysis design to develop a better comprehension of the perceptions that nursing students had when engaging in video-based remote simulation experiences. The approach employed in this research is based on Colaizzi's phenomenological theory, which draws on the participants' perspectives and impressions to describe the phenomenon at hand, resulting in the identification of similar characteristics within the study population rather than individual characteristics [8]. This paper uses a constructivist epistemology to examine the respondents' perceptions, bearing in mind that they are contextual, personal, and influenced by how they interpret the world around them and how they react to phenomena [9].

Setting and Sample

A purposive sample of nursing students from various academic levels at one public university in Saudi Arabia which receives students coming from different geographic, economic, and cultural backgrounds was employed in this study [10]. Maximum variation sampling technique was utilized to recruit an internally heterogenous and externally homogenous diverse sample, to tackle the diverse experiences of students. The students included were enrolled in the full-time study program, and had not engaged in remote video-based simulation before the COVID-19 pandemic. 39 students were invited to participate in this study, and 32 agreed to take part, thus resulting in a response rate of 82.05%. The students in this study took part in video-based simulation for the first time through courses such as health assessment, adult health nursing, and practicum nursing. In the nursing program at this nursing school, face-to-face high-fidelity simulation is usually used throughout the four academic years, starting in the first semester, however, video-based simulation was introduced for the first time.

Video-Based Simulation

During quarantine, this novel framework was implemented through Blackboard Collaborate Launcher, a virtual forum for online video meetings offered by the university. After face-to-face university lectures were canceled, all previously planned simulation experiences were revised and modified to the current condition. Six video simulation sessions were conducted with this method, simulating patients in multiple clinical circumstances. To resolve each simulation situation, students had to conduct nursing tasks similar to the required NIC (Nursing Interventions Classification) such as admission care, history taking, counseling, health education, and health promotion [11]. Every simulated clinical condition was conducted through video technology by consenting standardized patients at home during the COVID-19 quarantine and all simulation sessions were based on the circumstances faced during these times. These simulation scenarios included situations such as an elderly hypertensive bedridden patient with decubitus ulcer, a patient with a hypertensive crisis, a patient who is suffering from multiple epilepsy episodes, post-laparoscopic cholecystectomy, and a woman subject to domestic violence and suffering from severe anxiety attacks. Through the COVID-19 disease outbreak, all standardized patients who represented patients as per the Association of Standardized Patient Educators Standards of Best Practice were quarantined, so all of the basic questions they posed regarding proper treatment and safety strategies were tackled, regardless of the cause for each scenario. The simulation approach was implemented according to the Standards of Best Practice: Simulation of the International Nursing Association for Clinical Simulation and Learning (INACSL) [12]. Every phase of the simulation session was conducted in this manner: pre-briefing (development of an emotionally healthy educational environment), briefing (prior knowledge relating to the simulation situation) simulated scenario (conducting the simulation experience), and debriefing (evaluating and discussing the clinical performance throughout the simulated scenario). Despite the fact that the standardized patients varied during the various virtual cases, they were all clinical simulation instructors mimicking a clinical case. They were selected for their familiarity with this technique and were prepared to perform their roles in each simulated situation, guaranteeing that the simulation experience was as realistic and as accurate as possible. Each simulation session was around 30 minutes with an added 10 minutes for pre-briefing and debriefing respectively. Each scenario was developed by a group of three nursing faculty members who are experts in simulation as well as in various nursing fields.

Recruitment and Data Collection

The ESOMAR guidelines for online research were adopted. Students were approached by email after the researchers were given permission to a database of the university webmail pool. No spam emails were used, and each student received one invitation email. Students were forwarded a general request to partake in the study as well as a description of the research intent and were expected to send back an email explicitly giving their consent to take part. Following the induction of research subjects, the researchers invited them to engage in hour-long semi-structured interviews through Microsoft Teams (Microsoft Corporation, Redmond, USA).

Interviews

The interviews were performed by four researchers: two lecturers, an assistant professor, and an associate professor, who specialized in nursing and have been instructing for at least the past two years at a higher educational institution. The researchers did not take part in evaluating the students in the video-based simulation sessions and were currently not evaluating the students in any of the courses, thus avoiding any biases. All the researchers had experience in qualitative data collection and analysis. The interviews were conducted through video conferencing platforms, which enabled the researchers to perform interviews until data saturation was achieved. In order for them to be accessible to offer accurate reports of their interactions, an appropriate time for the interviews was decided upon with each person, particularly given their busy academic calendars. To prevent the possibility of a moderator's dominance, the researchers conducted the interviews in alternation. The following questions were posed, “How do you describe your experience with virtual video-based simulation in substitution of face-to-face simulation experiences?”, “How do you describe the enablers and barriers of participating in the virtual video-based simulation experiences?”, “How do you describe the advantages and drawbacks of such an experience?”.

Thematic Analysis

The participants consented that the online interviews would be screen-recorded in order to maintain the data and analyze it. The participants' reports were transcribed in English and interpreted using an inductive thematic content analysis, which involved narrating the participants' verbatim accounts, open text marking, and creating themes [13]. A professional sworn translator who had a background in public health was contacted and the blinded transcripts were translated into English and then back-translated into Arabic to make sure of the accuracy of the verbatim. Each researcher then performed their own evaluation, and then the researchers gathered and debated the findings until they came to a consensus on the emerging conclusions, while avoiding taking their personal viewpoints into the conversation. The quotes were given a descriptive and insightful term that illustrates the actual essence of the datum given, and then those words were clustered, reorganized, and collated into qualitative themes, which were carefully considered by the researchers to ensure a thorough and accurate understanding of the students' narratives.

Trustworthiness and Credibility

Various protocols were conducted by the authors in compliance with prior evidence in the area of qualitative analysis in order to enhance the trustworthiness of the research and prevent biases from arising [14]. Concurrent data analysis ensured that the themes generated could be thoroughly explored by concurrent interviews for a full interpretation of the phenomena. All of the researchers used the same questioning structures and asked the same questions, and they made sure to thoroughly examine any emerging ideas and avoid having any blind spots in any of the results. To express the research findings, several quotes were used, giving the participants in this study a true voice. Specialists of qualitative analysis were also contacted, and the findings were double-checked using a control protocol. For instance, the consulted experts reviewed the alignment of the transcripts, codes, generated themes, and quotations to provide an external perspective into the quality of the analysis process. The authors used member-checking to ensure reliability and validity, with the themes being forwarded to the respondents for confirmation after they were finalized [15]. Furthermore, external member-checking was utilized to guarantee that the findings represent the population at hand, in which students who have resembling characteristics to those who participated in the analysis were consented and asked to consider the parallels between the emergent themes and their own experiences. These students were from the same classes the participants were in, but were not able to take part in the study at the time of data collection but were able to give their honest opinion on the results afterward.

Ethical Considerations

The researchers obtained permission from King Saud University's Research and Ethics Committee (ECO-R-80). The study's aims were already clarified to respondents, and all of them sent informed consent via email to take part in the research. The students were clearly informed that participating is strictly voluntary and confidential, and any identifying data would not be noted in data collection or analysis. This project was conducted in accordance with the international Declaration of Helsinki's principles and guidelines.

## Results

Results

Sample Characteristics

The sample of this study comprised 32 nursing students from one nursing school in Saudi Arabia, where eight (25%) of them were first year, seven (21.88%) were second year, seven (21.88%) were third year and 10 (31.25%) were fourth-year nursing students (Table 1).

**Table 1:** Participant Characteristics

Variable	Category	N	%
Academic year	First Year	8	25
	Second Year	7	21.88
	Third Year	7	21.88
	Fourth Year	10	31.25

Phenomenology

The qualitative analysis has given rise to five main themes: “Enhanced Learning and satisfaction”, “Improved communication skills”, “Lack of hands-on experience”, “More comfortable experience”, and “Technical Barriers” (Table 2).

**Table 2:** Thematic Tree

Theme	Verbatim	Code	Participant
Enhanced Learning and satisfaction	“…video-based simulation experiences helped me get back into the spirit of nursing practice and nurse-patient interaction…”	Helped me	S8
	“…Video-based simulation is a totally different and rich dimension that helped me improve my nursing abilities…”	Improved	S13
Improved communication skills	“…I was able to pay attention to certain pitfalls in my communication and I wouldn’t have been able to discern them in regular scenarios…”	Pay, attention communication	S32
	“… now I can definitely communicate proficiently with patients without anxiety or hesitation…”	Now I can communicate	S20
Lack of hands-on experience	“…I am worried if we did not go to the clinical setting anytime soon we would lose the technical dexterity…”	Lose technical dexterity	S28
	“…I need the opportunity to actually apply what I learned with an actual patient…”	Apply, actual patient	S30
More comfortable experience	“… I did not feel the frustration or anxiety I used to feel in regular simulation…”	Not feel, anxiety	S19
	“…more focus while you do not have direct view of the evaluators…”	More focus	S10
Technical Barriers	“…it was very frustrating to deal with the slow internet connection…”	Slow, connection	S1
	“…The internet connection would sometime go down and it would make the patient interview really challenging…”	Internet connection	S29

Enhanced Learning and Satisfaction

The nursing students who took part in this study were asked to express their perceptions regarding their experience with video-based simulation instead of face-to-face mannequin-based simulation. The students conveyed that in light of the current situation and after the suspension of clinical practice, video-based experience filled the gap and improved their learning, which made them feel more content regarding their training. For instance, one of the students said, “The coronavirus pandemic and for months has disabled our clinical preparation, we went through an intense phase of confusion regarding our practice…video-based simulation experiences helped me get back into the spirit of nursing practice and nurse-patient interaction… I feel compensated and I am learning valuable information regarding patient cases…” (S8). Another student also indicated, “…these experiences do fill some gap regarding clinical practice as we get to be exposed to different patient cases where we can practice health promotion and the nursing process… it has enriched my learning on various levels and mainly helped me focus on certain information that I wouldn’t have been able to focus on in regular cases…” (S22). Further account was also shared by one of the students, “… I did not expect it to be as valuable as regular simulation or clinical practice, but I was surprised about its significance… video-based simulation is a totally different and rich dimension that helped me improve my nursing abilities and critical thinking… I feel very excited about further experiences …” (S13).

Improved Communication Skills

Another theme that was emphasized by all the students through the semi-structured interviews was that video-based simulation experiences enhanced the students’ communication skills with patients, as it isolated the nursing interventions emphasizing on communication-based techniques. For instance, one of the students proclaimed, “… usually in clinical practice and in regular simulation I tend to focus on the technical skill itself, while in video-based simulation I got to focus on choosing my words properly, how to address the patient, and how to provide the patient with educational intervention…” (S32). Another student had a similar experience, “… I was able to pay attention to certain pitfalls in my communication and I wouldn’t have been able to discern them in regular scenarios… this experience was the perfect opportunity to work on advancing my communication skills…” (S9). Another anecdote was, “… now I can definitely communicate proficiently with patients without anxiety or hesitation… I know exactly what to say and when to say it…” (S20).

Lack of Hands-On Experience

Regarding the disadvantages of video-based simulation, the students expressed their worries regarding the lack of hands-on experiences where they might lose their technical nursing skills due to disruption of practice. For example one of the testimonies was, “… I know it is quite beneficial… I have been learning a lot but I am worried if we did not go to the clinical setting anytime soon we would lose the technical dexterity and proficiency in carrying out nursing procedures…” (S28). Another student also said, “… I can’t remember the last time I inserted an IV cannula or I drew a blood sample or suctioned a patient… I feel like the techniques are quite blurry and I need practice to gain back the skills… I mean nursing is not only about carrying out such procedures, but it is quite important and I want to graduate proficient enough to be inducted into professional practice…” (S3). A comparable experience was also shared, “…I am enjoying the video simulations but I can’t wait to get back to clinical practice as I have learned a lot during these experiences, yet I need the opportunity to actually apply what I learned with an actual patient in the hospital and combine these very important skills with the technical and hands-on skills that I need to master as a registered nurse…” (S30).

More Comfortable Experience

Another prevalent theme that was expressed by the students indicated that they have experienced more comfort while performing in video-based simulation scenarios than in regular mannequin-based simulation-based scenarios. The students have related this to the advantage of having an actual person responding to them while carrying out their care rather than having an inanimate doll. For instance, one of the students has expressed, “… I felt more comfortable implementing the interventions on a living responsive patient… the patient helped me feel more competent and professional and the fact that I was behind a screen took the edge off a little bit, especially that the evaluators in video-based simulation were kind of hidden so it eased frustration…” (S27). Another student also shared, “… I did not feel the frustration or anxiety I used to feel in regular simulation… it was kind of smoother and easier to cope with… I felt more confident and more asserted…” (S19). A similar account was also shared, “… usually in regular simulation there are a lot of details that you have to keep in mind especially while carrying out hands-on procedures and having the evaluators watching you on every step you make, however in video-based simulation you have the chance of more focus while you do not have direct view of the evaluators which gives a feeling of security and less anxiety of being watched…” (S10).

Technical Barriers

The final theme that was indicated throughout the semi-structured interviews by some students related to the technical barriers that came with participating in an online video-based simulation. The students have expressed their struggle with internet connection sometimes and with operating system issues of their laptops some other times which led to their inability to attend their assigned sessions on various occasions. For example, one of the accounts was, “…it was very frustrating to deal with the slow internet connection… the video conference would lag or it would get cut off multiple times and I would have to miss some information or have the patient repeat the history taking multiple times… it was messy…” (S1). Another student also proclaimed, “… one time the operating system of my laptop would not launch I missed almost 20 minutes of the simulation session and I had to skip through questions and keep the patient waiting… until I signed in there was a lot of frustration…” (S17). A similar anecdote was also shared, “…it is quite an interesting experience but the technical issues make it difficult to stay on top of it… The internet connection would sometimes go down and it would make the patient interview really challenging…” (S29).

## Discussion

The COVID-19 pandemic altered the practice environment among both healthcare providers and healthcare educators, where video interventions are now regarded as the future of healthcare [16]. As a result, this research project believes that, despite the limited adjustment of clinical simulation practices for students due to university closures during the pandemic, teaching and preparing nursing students in this method of healthcare is critical to adapting them to modern healthcare requirements.

The results of this study have shown through the thematic analysis that the nursing students positively expressed being satisfied with video-based simulation experiences where they reported themselves to be learning new information and improving their critical thinking skills and communication skills. The students reported that they were being exposed to new patient cases and were able to analyze data more intricately and communicate more proficiently, thus practicing a different aspect of nursing care through communication. This is consistent with Jiménez-Rodríguez and Arrogante who have found that the students' remarks, which illustrated their immense satisfaction with and appreciation of the procedure and its adjustment of the model for conducting clinical simulation interventions during the interruption of in-classroom practices, affirm the findings of high satisfaction with virtual video simulations [17]. Another benefit mentioned was the ability to learn from mistakes and exercise in a practical setting during the simulations performed. Our results were also in line with Yeun et al. which found that nursing students placed a high emphasis on the knowledge gained from virtual video simulations because they believed it would be useful in their eventual clinical practice [18]. In this regard, Hollander and Carr noted that the pandemic posed a challenge to health systems around the world, resulting in an increase in the usage of telemedicine services, especially video consultations [6]. In support of our results, Portnoy et al. have indicated that to effectively handle a video consultation and deliver high-quality patient care, learning in this modality of health care is needed [19]. Our findings are also in line with Bracq et al., who found that the students also believed that this modality aided in the development and/or reinforcement of non-technical abilities such as communication, active listening, appearance, empathy, and teamwork [20]. While further research is needed in this area to evaluate the growth of non-technical skills across virtual simulation modalities, clinical simulation is also proven to help with this [21].

The results of our study showed that the nursing students in this study have experienced more comfort and convenience in comparison to face-to-face regular simulation, where they have reported feeling more comfortable and asserted and less intimidated. This is inconsistent with Jiménez-Rodríguez and Arrogante who have found that the participating students have reported experiencing lower levels of stress during regular simulation sessions while they indicated to be more anxious in video-based simulation [17]. However, our findings are in line with Cantrell et al., who have shown that nursing students tend to be more comfortable in video-based simulations where they feel more secure [22].

Furthermore, the nursing students in our study have indicated that video-based simulation raised their concerns regarding the maintenance of their nursing skills in carrying out technical procedures. The students also conveyed their frustration regarding technical difficulties that they have faced while participating in these simulation sessions. This is consistent with Jiménez-Rodríguez and Arrogante who have shown that students experienced technical difficulties that have hindered their effective participation in video-based simulations, where they also felt that it was not effective in improving their technical abilities in nursing practice [17]. These drawbacks were very similar to those mentioned by healthcare practitioners in real-time video consultations, who often expressed dissatisfaction with their failure to conduct physical examinations and therapeutic techniques or procedures. In this regard, it's worth noting that some therapeutic practices are now being adapted using new platforms and technology [23].

Our results resonate with findings that indicate that simulation-based learning enhanced a sense of confidence, performance, and efficiency among nursing students [24]. This is also in line with a recent study showing that video-based learning enhanced these same outcomes [25].

Limitations

This study gave us an insight into the students’ experience with video-based simulation, which is a first step into understanding its benefits in the undergraduate nursing program. However, this study does not validate the implication that the students gained knowledge to enhance their clinical practice as this study is qualitative, thus a longitudinal quantitative study is needed to test out the method’s impact on the students’ learning outcomes.

## Conclusions

In response to the challenges posed by the COVID-19 pandemic, restructuring and rethinking clinical education in nursing is key to filling the gap between theory and practice nowadays as well as preparing nursing education to be flexible in adapting to severe situations that might restrict its practicality. In response to the simulation-based education demands generated by the COVID-19 epidemic, a novel simulation approach that reproduces high-fidelity experiences using virtual video-based scenarios designed around specific activities and practices for nursing care is proposed. A qualitative inquiry was pertinent due to the possibility of a diverse perception of the learning process in the context of an interactive new learning modality. The students in this study indicated that they were satisfied with video-based simulation, where they reported enhanced learning, better communication skills, and more perceived comfort. The drawbacks of this modality were related to technical barriers and lack of hands-on experience in technical procedures which are challenging to deliver through video. Virtual video-based simulation may be seen as an additional source of learning to be used in conjunction with in-person training rather than an alternative source of learning altogether. It can be most efficient for communication-based skills not only in the present COVID-19 scenario but could also be applied to other circumstances. This study is merely the stepping stone for future research that should use a longitudinal quantitative design in order to test out the method’s impact on students’ learning outcomes.
